# Spatial Variation and Resuscitation Process Affecting Survival after Out-of-Hospital Cardiac Arrests (OHCA)

**DOI:** 10.1371/journal.pone.0144882

**Published:** 2015-12-14

**Authors:** Chien-Chou Chen, Chao-Wen Chen, Chi-Kung Ho, I-Chuan Liu, Bo-Cheng Lin, Ta-Chien Chan

**Affiliations:** 1 Center for Geographic Information Science, Research Center for Humanities and Social Sciences, Academia Sinica, Taipei, Taiwan; 2 Division of Trauma, Department of Surgery, Kaohsiung Medical University Hospital, Kaohsiung, Taiwan; 3 Department of Emergency Medicine, Kaohsiung Medical University Hospital, Kaohsiung Medical University, Kaohsiung, Taiwan; 4 Department of Health, Kaohsiung City Government, Kaohsiung, Taiwan; 5 Fire Bureau, Kaohsiung City Government, Kaohsiung, Taiwan; Azienda Ospedaliero-Universitaria Careggi, ITALY

## Abstract

**Background:**

Ambulance response times and resuscitation efforts are critical predictors of the survival rate after out-of-hospital cardiac arrests (OHCA). On the other hand, rural-urban differences in the OHCA survival rates are an important public health issue.

**Methods:**

We retrospectively reviewed the January 2011–December 2013 OHCA registry data of Kaohsiung City, Taiwan. With particular focus on geospatial variables, we aimed to unveil risk factors predicting the overall OHCA survival until hospital admission. Spatial analysis, network analysis, and the Kriging method by using geographic information systems were applied to analyze spatial variations and calculate the transport distance. Logistic regression was used to identify the risk factors for OHCA survival.

**Results:**

Among the 4,957 patients, the overall OHCA survival to hospital admission was 16.5%. In the multivariate analysis, female sex (adjusted odds ratio:, AOR, 1.24 [1.06–1.45]), events in public areas (AOR: 1.30 [1.05–1.61]), exposure to automated external defibrillator (AED) shock (AOR: 1.70 [1.30–2.23]), use of laryngeal mask airway (LMA) (AOR: 1.35 [1.16–1.58]), non-trauma patients (AOR: 1.41 [1.04–1.90]), ambulance bypassed the closest hospital (AOR: 1.28 [1.07–1.53]), and OHCA within the high population density areas (AOR: 1.89 [1.55–2.32]) were positively associated with improved OHCA survival. By contrast, a prolonged total emergency medical services (EMS) time interval was negatively associated with OHCA survival (AOR: 0.98 [0.96–0.99]).

**Conclusions:**

Resuscitative efforts, such as AED or LMA use, and a short total EMS time interval improved OHCA outcomes in emergency departments. The spatial heterogeneity of emergency medical resources between rural and urban areas might affect survival rate.

## Introduction

Out-of-hospital cardiac arrest (OHCA) is a critical and emergent threat for public health around the world. In the United States [1] and Europe [2], around 300,000 persons experience an OHCA each year, and the mortality rate is high (approximately 90%). To enhance the survival rate and improve the neurological outcomes after cardiac arrest are the challenges for emergency medical services (EMS) and the public.

Predictors of survival after OHCA have been systematically investigated [3,4]. Factors affecting OHCA survival can be classified into spatial and non-spatial factors. The non-spatial factors were: (1) patients’ characteristics, such as age [5], sex [6], and comorbidity [7]; (2) OHCA etiology, such as trauma injury [8–10]; (3) resuscitation process: bystander and EMS cardiopulmonary resuscitation (CPR) [3], using an automated external defibrillator (AED) onsite [11], and advanced airway management, such as laryngeal mask airway (LMA) [12].

On the other side, spatial factors included response time [13,14], transport distance [15], population density [16], and rural-urban differences [17]. The first two factors were directly correlated to the EMS time interval from the call to hospital admission. A short EMS response time has been reported to enhance the survival rate [13]. The latter two factors were considered to represent the spatial heterogeneity within each area. Furthermore, the spatial distribution of emergency hospitals and the density of EMS coverage were also affected by the underlying socio-economic environment. Thus, while attempting to improve the OHCA survival rate, both spatial and non-spatial factors must be considered simultaneously. To the best of our knowledge, less is known about the relative importance of these factors. With in-depth understanding of these factors, we might have the chance to strengthen the links in the chain of survival [18].

In this study, we elucidated how spatial and non-spatial factors simultaneously affect post-OHCA survival until hospital admission. In addition to identifying and quantifying the possible risk factors, the geo-visualization of the spatial factors was also found to be beneficial for future EMS allocation planning.

## Materials and Methods

### Ethics

The study was approved by the committee of the institutional review board (IRB) at Academia Sinica (AS-IRB01-14013). The databases we used were all stripped of identifying information and thus informed consent of participants was not required.

### Study design and settings

This was a three-year retrospective study of a cardiac arrest cohort in the City of Kaohsiung, Taiwan. Kaohsiung spans 2,947 km^2^ and includes both urban and rural communities. The total population of Kaohsiung is 2.77 million in 2015. The four levels of designated acute care hospitals in Taiwan, from the lower level to the higher level, are district hospital (levels 0, 1), regional hospital (level 2), and medical center (level 3: tertiary medical service). Kaohsiung has 25 acute care hospitals, including four medical centers, responsible for emergency management of all OHCA cases. The records of OHCA patients were obtained from the Kaohsiung City Department of Health. We enrolled all patients who suffered an OHCA and were treated by emergency medical technicians (EMTs) between January 1, 2011 and December 31, 2013. Cardiac arrest was defined as the abrupt loss of heart function in a person as confirmed by EMTs. Since the discharge data was unavailable, we evaluated the predictors’ influence on OHCA patients’ emergency department (ED) outcomes. The primary outcome measure was the overall post-OHCA survival until hospital (intensive care unit) admission.

The OHCA data was based on the international Utstein-style criteria and comprised demographic information of a patient, witness status, EMS response time (the interval between the ambulance departure from the fire station to the ambulance arrival at the scene), EMS time at the scene (the interval from the ambulance arrival at the scene to the ambulance departure from the scene), EMS transport time (the interval from the ambulance departure from the scene to the ambulance arrival at the hospital), type of cardiovascular life support used, name of the destination hospital, name of the dispatch unit, as well as location of the cardiac arrest. In addition, data regarding the four levels of designated acute care hospitals and their corresponding addresses were acquired from the Kaohsiung City Department of Health.

### Data processing

**Fig 1:**
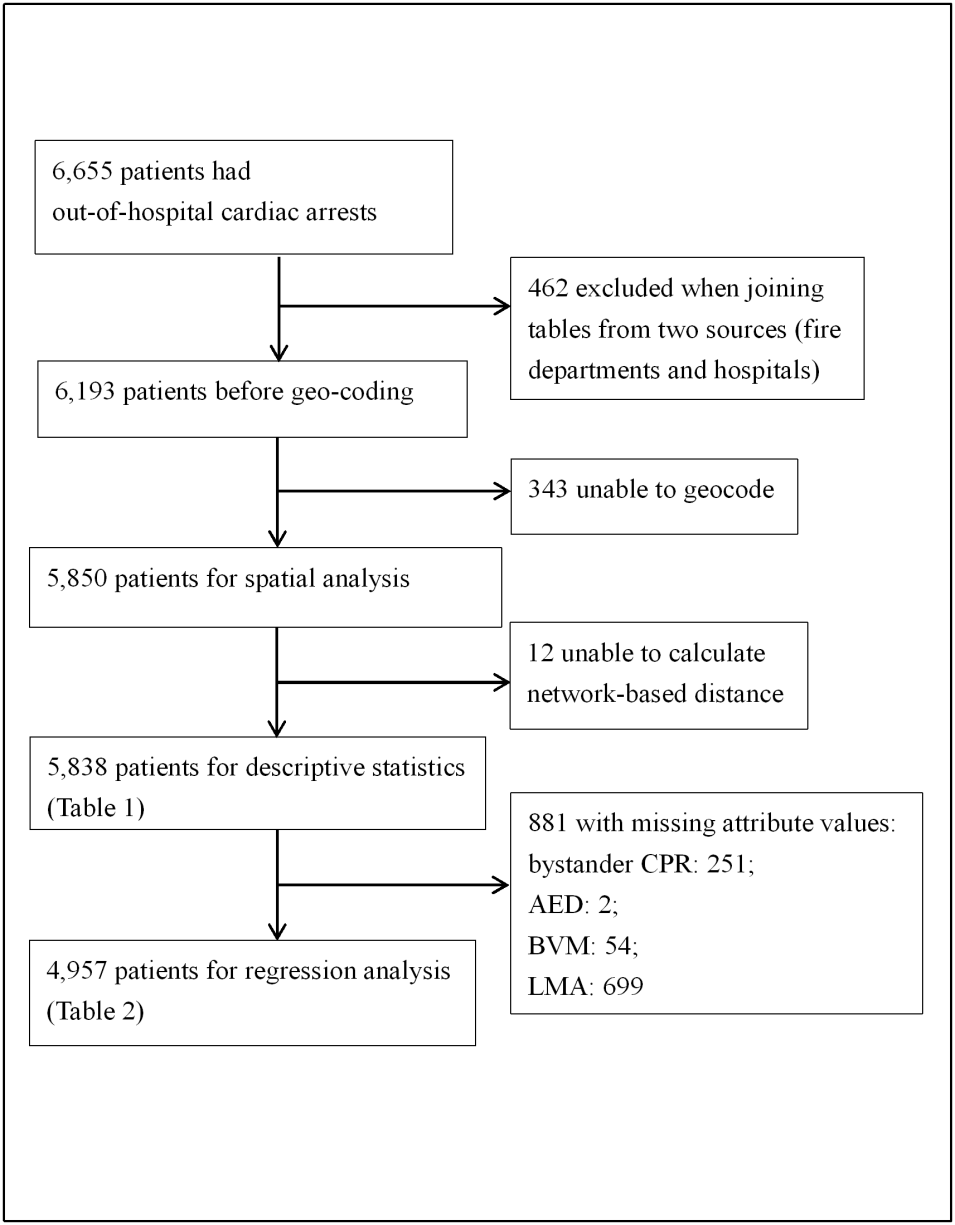
Flow chart of the study design. Abbreviations: CPR: cardiopulmonary resuscitation; AED: automated external defibrillator; LMA: laryngeal mask airway.

OHCA data were recorded separately from two sources: the City’s Fire Department and destination hospitals. Using the name of the dispatch unit and the time of hospital admission as primary keys, 6,193 of 6,655 (93.1%) observations were merged successfully (Fig 1). Because of missing addresses, we excluded 343 observations (5%) which were unable to geocode. Among the 5,850 observations eligible for network analysis, 12 were isolated from the nearby street network. These observations were beyond the threshold of tolerance distance and hence were excluded while calculating the driving distance. We further excluded 881 cases with missing attribute values and the final observations for regression analysis were 4,957.

### Geospatial analysis

The driving distance to the hospital after an OHCA event was calculated using ESRI’s network analysis extension in ArcGIS 10.2 (ESRI Inc., Redlands, CA, USA). Patients’ addresses or locations of arrest were geocoded by using the position service provided by the Ministry of the Interior, Taiwan. To protect patients’ privacy, a GeoMasker tool [19] was applied and the precision of each case’s coordinate was given 10 meters’ tolerance. Further, a geo-statistical method, Kriging [20,21], was used to generate an estimated surface. This surface represented the interpolation of the time interval from the call to hospital admission. Kriging assumes that the distance between the origin and the endpoint of cardiac arrest reflects a spatial correlation that can be used to explain variation in the surface.

**Fig 2:**
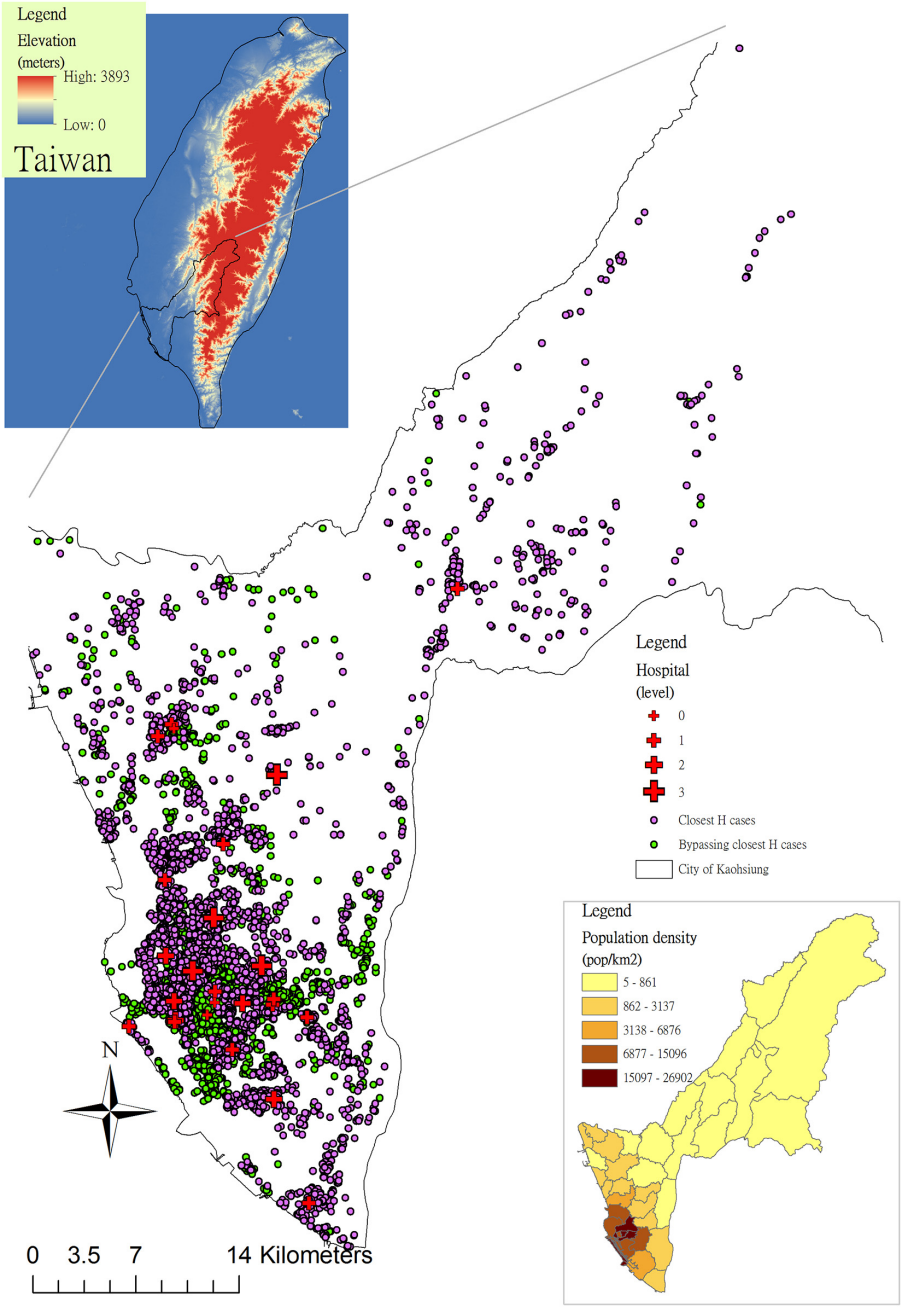
Study areas, population densities, and distributions of out-of-hospital cardiac arrests (OHCA) cases and hospitals in Kaohsiung, 2011–2013. Purple points indicate that patients with OHCA were transported to the closest emergency hospital while green points represent bypassing it.

Population densities in 2013 and the survival rates during 2011–2013 in 38 districts were analyzed and displayed by ArcGIS. Eastern Kaohsiung has hilly areas where the population densities are lower than in the plains (Fig 2). We used digital elevation data to represent the elevation variations, and the data were downloaded from the ASTER GDEM website (http://gdem.ersdac.jspacesystems.or.jp/search.jsp), which is a publicly available dataset for global digital elevation with a 30-meters resolution [22].

### Statistical analysis

Univariate and multivariate logistic regressions were applied to evaluate the association between risk factors and survival after OHCA. Odds ratios (ORs) and their 95% confidence intervals (CIs) were estimated after adjustment for confounding factors. Age (< = 65, > 65 years old), sex, home or public areas (street, work, sports facility, and public building), bystander witness (yes, no), bystander CPR (yes, no), AED shock (with or without shock during prehospital EMT treatment), LMA use (yes, no), trauma (yes, no), type of emergency hospital (medical center, non-medical center), bypassed the closest hospital (yes, no), cardiac arrest occurred in high population density areas (> = 5,000 persons/km^2^) (yes, no), interval from the call to hospital admission (min), and network-based transport distance (km) were analyzed. All variables were categorical except for interval from the call to hospital admission (i.e., total EMS time interval) and transport distance. Statistical analyses were performed using SAS 9.4 statistical package (SAS, Cary, USA). All tests were two-tailed, and p values less than 0.05 were regarded as significant.

### Results

Among the 5,838 patients who were eligible for descriptive statistics analysis, 961 (16.5%) survived until hospital admission (Fig 1). The remarkable difference was observed in survival percentages between the high and low population density areas (9.8%, p < 0.001 in Table 1). On average, the interval from the call to hospital admission and the transport distance were 20.4 min (SD = 8.0 min) and 3.9 km (SD = 5.1 km), respectively (Table 1).

**Table 1:** Characteristics of patients with out-of-hospital cardiac arrests in Kaohsiung, 2011–2013.

Variable	Subgroup	Total (*n* = 5,838)	Death (*n* = 4,877)	Survival (*n* = 961) (%)	p value*
Age	< = 65	2,566	2,138	428 (16.6)	0.695
	>65	3,272	2,739	533 (16.2)	
Sex	Male	3,727	3,151	576 (15.4)	0.006
	Female	2,111	1,726	385 (18.2)	
Home	Yes	4,652	3,904	748 (16.0)	0.124
	No	1,186	973	213 (17.9)	
Bystander CPR (missing = 251)	Yes	832	679	153 (18.3)	0.083
	No	4,755	3,997	758 (15.9)	
AED shock (missing = 2)	Yes	392	293	99 (25.2)	< .001
	No	5,444	4,583	861 (15.8)	
LMA use (missing = 699)	Yes	2,087	1,673	414 (19.8)	< .001
	No	3,052	2,628	424 (13.8)	
Trauma	Yes	668	582	86 (12.8)	0.007
	No	5,170	4,295	875 (16.9)	
Emergency hospital level	Medical center	1,737	1,471	266 (15.3)	0.132
	Non-medical center	4,101	3,406	695 (16.9)	
Bypassed the closest hospital	Yes	2,374	1,946	428 (18.0)	0.007
	No	3,464	2,931	533 (15.3)	
High population density areas**	Yes	3,632	2,900	732 (20.1)	< .001
	No	2,206	1,977	229 (10.3)	
Mean time from the call to hospital admission (min)	-	20.4 (SD = 8.0)	20.6 (SD = 8.0)	18.9 (SD = 7.7)	-
Mean transport distance (logarithm of km)	-	3.9 (SD = 5.1)	4.0 (SD = 5.1)	3.3 (SD = 4.9)	-

Abbreviations: CPR: cardiopulmonary resuscitation; AED: automated external defibrillator; LMA: laryngeal mask airway.

*Equal proportion (χ2) tests on survival by subgroup.

**> = 5,000 persons/km^2^.

**Fig 3:**
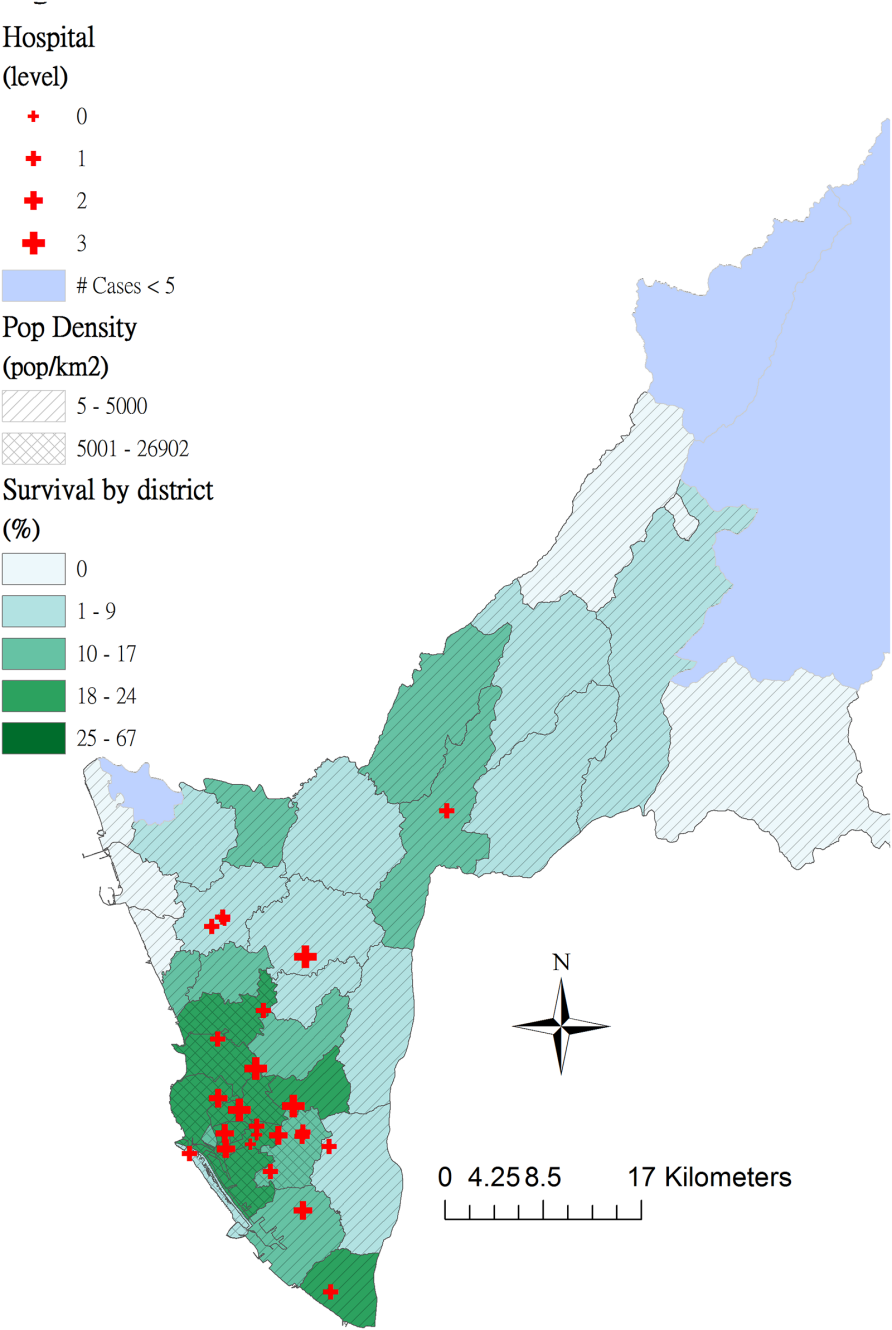
Survival percentages of out-of-hospital cardiac arrests (OHCA) by district in areas with high (> = 5,000 persons/km^2^) and low (< 5,000 persons/km^2^) population densities of Kaohsiung City, 2011–2013.

The distribution of patients with OHCA was proportional to that of population in the City of Kaohsiung (Fig 2). Dots of OHCA cases were concentrated in the South-western metropolitan areas of Kaohsiung. Compared to green points (ambulance that bypassed the closest hospital), purple points (patients were transported to the closest hospital) increasingly clustered around high-level emergency hospitals. Survival percentages after OHCA by district revealed spatial heterogeneity (Fig 3). In the high population density areas (> = 5,000 persons/km^2^), the survival percentage was high and vice versa.

Multivariate logistic regression analysis showed that the female sex (adjusted odds ratio, AOR, = 1.24, 95% CI = [1.06–1.45]), OHCA events in public areas (AOR = 1.30, 95% CI = [1.05–1.61]), exposure to AED shock (AOR = 1.70, 95% CI = [1.30–2.23]), LMA use (AOR = 1.35, 95% CI = [1.16–1.58]), non-trauma (AOR = 1.41, 95% CI = [1.04–1.90]), bypassed the closest hospital (AOR = 1.28, 95% CI = [1.07–1.53]), and within the high population density areas (AOR = 1.89, 95% CI = [1.55–2.32]) were associated with improved post-OHCA survival until hospital admission (Table 2). However, a prolonged resuscitation period from the call to hospital admission was associated with high OHCA mortality (AOR = 0.98, 95% CI = [0.96–0.99]).

**Table 2:** Factors contributing to survival of patients with out-of-hospital cardiac arrests until hospital admission in Kaohsiung, 2011–2013.

Factors	Univariate		Multivariate	
	Odds ratio (95% CI)	p value	Odds ratio (95% CI)	p value
Age > 65 (vs. < = 65)	0.96 (0.82–1.12)	0.611	0.91 (0.77–1.07)	0.274
Female (vs. Male)	1.19 (1.02–1.39)	0.022	1.24 (1.06–1.45)	0.007
Public area (vs. home)	1.19 (0.99–1.43)	0.060	1.30 (1.05–1.61)	0.014
Bystander CPR (vs. no bystander CPR)	1.14 (0.92–1.40)	0.215	1.01 (0.81–1.26)	0.892
AED shock(vs. no AED shock)	1.86 (1.44–2.41)	< .001	1.70 (1.30–2.23)	< .001
LMA (vs. no LMA) use	1.52 (1.30–1.77)	< .001	1.35 (1.16–1.58)	< .001
Non-trauma (vs. Trauma)	1.37 (1.05–1.78)	0.019	1.41 (1.04–1.90)	0.022
Medical center (vs. non-medical center)	0.90 (0.76–1.06)	0.236	0.84 (0.70–1.00)	0.059
Bypassed the closest hospital (vs. closest hospital)	1.28 (1.10–1.49)	0.001	1.28 (1.07–1.53)	0.005
High population density areas*(vs. low)	2.25 (1.89–2.69)	< .001	1.89 (1.55–2.32)	< .001
Time from the call to hospital admission (min)	0.96 (0.95–0.98)	< .001	0.98 (0.96–0.99)	0.002
Transport distance (logarithm of km)	0.84 (0.77–0.91)	< .001	0.98 (0.87–1.10)	0.751

Abbreviations: CPR: cardiopulmonary resuscitation; AED: automated external defibrillator; LMA: laryngeal mask airway.

*> = 5,000 persons/km^2^.

**Fig 4:**
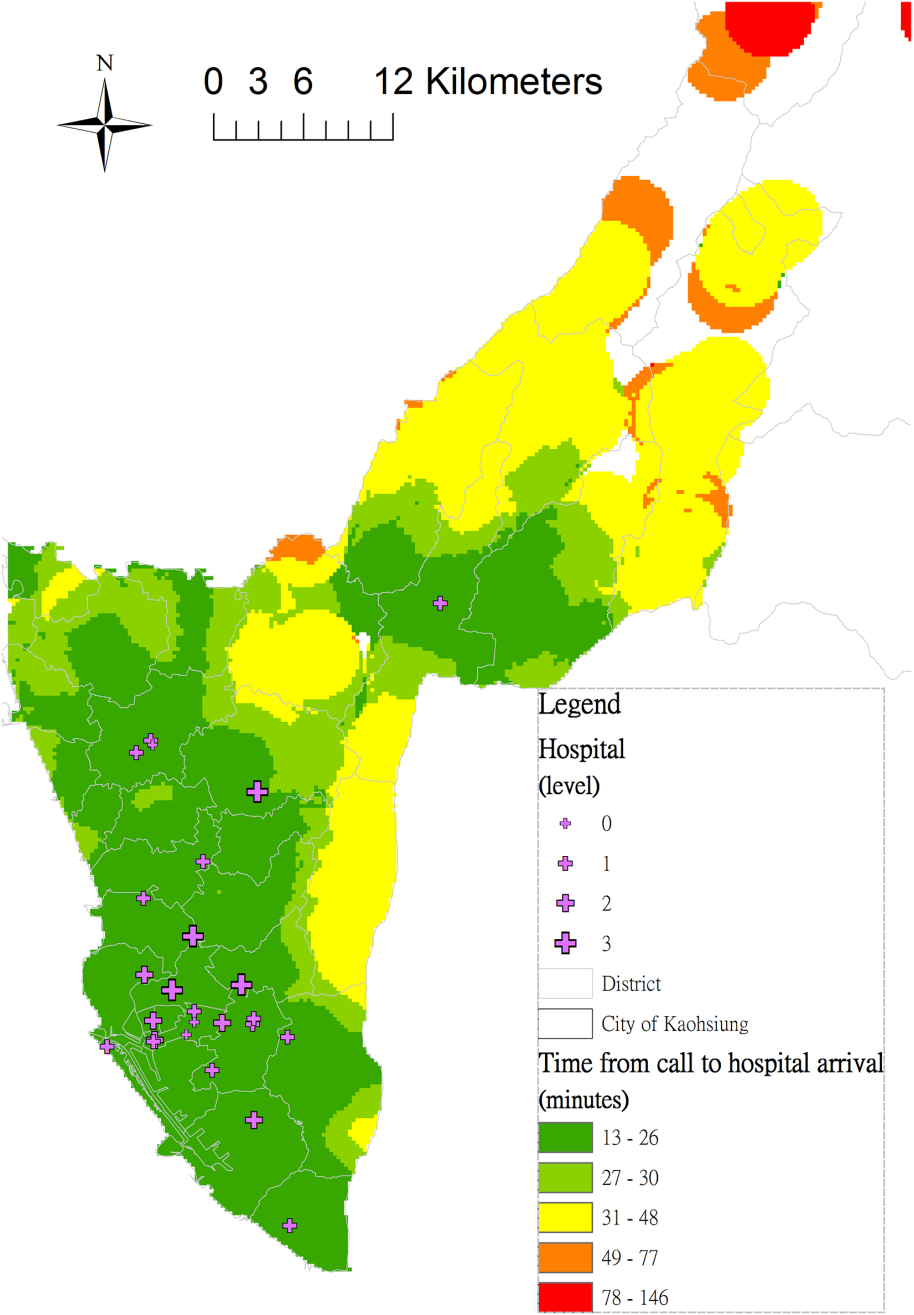
Interpolation of the interval from the call to hospital admission by using the Kriging method. Areas marked with red indicate a prolonged interval of an out-of-hospital cardiac arrest (OHCA) case from the call to hospital admission, while areas marked with green show a short interval (<30 min). Uncolored areas are locations without sufficient interpolation information (sparse OHCA event points).

The interpolated map (Fig 4) generated using the Kriging method revealed that as an increased distance from the emergency hospital, the interval from the call to hospital admission naturally grows. Areas with a prolonged interval (> = 30 min) were: (1) the North-eastern hilly areas (orange and red); (2) the suburban areas of Kaohsiung (yellow).

## Discussion

In this three-year cohort study, the OHCA survival rate was 16.5% in a metropolis in southern Taiwan. After risk adjustment, female sex, events in public areas, exposure to AED shock, LMA use, non-trauma events, bypassed the closest emergency hospital, and high population density areas were positively associated with improved post-OHCA survival until hospital admission, whereas the interval from the call to hospital admission was negatively associated with OHCA survival.

Our study identified certain non-spatial risk factors, which were consistent with those reported in previous studies, such as the female sex, non-trauma, exposure to AED shock, and LMA use. Among these risk factors, AED has been proven to improve outcomes of patients with OHCA [23,24]. Likewise, the outcomes of traumatic cardiac arrest have been notoriously dismal [25]. Taiwan researchers have found that OHCA children with trauma had a lower chance of survival than non-trauma children [26]. Improved OHCA outcomes in females were observed in our study, which has been increasing reported in recent years [27,28]. Influences of sex and estrogen on OHCA outcomes require further exploration. Furthermore, we also found that LMA use was beneficial for patients with OHCA. The use of LMA for airway management by trained personnel during OHCA resuscitation is a feasible and beneficial method for effective airway management [29]. Currently, Kaohsiung has a shortage of the highest level of paramedics; therefore, all EMTs are trained to insert an LMA instead of an endotracheal tube to secure the airway in prehospital settings.

Regarding the spatial variables, OHCA occurring at home was an independent risk factor for poor survival in the present study. Studies on arrest location have yielded contrasting results. A Singaporean study reported that location alone had no independent effect (AOR = 1.13, 95% CI = [0.32–4.05]) on survival [30]. However, researchers from New Zealand and the Netherlands have concluded that OHCA survival in public locations was higher than that at homes [31,32]. Weisfeldt et al. also found that the rate of survival to hospital discharge in OHCA with initial shockable rhythms was 34% in public locations versus 12% at home in 10 North American communities [33]. The inconsistency in the reported results reflects the difficulty of evaluating OHCA in a multifactorial environment. Bystanders witnessing cardiac arrest events at home might play crucial roles in resuscitative efforts during OHCA [3]. However, bystander’ presence at the scene of at-home OHCA is closely related to family components, housing patterns or community systems, which might be highly discrepant across regions within a county, or across other countries [34]. It would be incorrect to extrapolate results without considering the regional variation in OHCA outcomes, which have been previously disclosed in only a limited number of studies [35,36]. We suggest that the present results are a step toward exploring more tailored strategies for regions with similar community infrastructure.

According to the Regulations for Emergency Medical Services of the study region [37], a patient with OHCA shall be transported to the designated hospital responsible for acute care or another appropriate nearby ED accredited by the government (Article 5). The surprising result that bypassing the closest emergency hospital increases the post-OHCA survival (AOR = 1.28, 95% CI = [1.07–1.53]) does not comply with the current EMS policy in Taiwan. In the remote areas of Kaohsiung where the total EMS time interval was longer (22.0~25.7 min in Table 3) than in the urban areas (17.5~20.0 min), most OHCA patients (*n* = 1,447) were transported to the closest emergency hospital. However, the chance of survival was relatively lower (mean of survival by district = 10.5%, SD = 7.5%) in the rural areas than in the urban areas (mean of survival = 20.2%, SD = 2.7%) (Fig 3). On the other hand, access to medical services in the metropolitan areas was not a major concern because of the spatial agglomeration of emergency hospitals. Therefore, the effect of total EMS time interval on OHCA survival in the urban areas of Kaohsiung was diluted and bypassing the closest hospital had a high survival rate of 21.73% in the subgroup analysis (Table 3). Similar studies have been reported in the United States, where patients transported to the closest hospital had poor survival (AOR = 0.82, 95% CI = [0.69–0.97]) compared to those transported to a distant hospital [15]. Nevertheless, the term “appropriate nearby ED” is not clearly defined by the government. Therefore, the hospital destination decision might be compromised between patients’ family members and EMTs and warrants further study.

**Table 3:** Survival rates and mean time intervals from the call to hospital admission according to population density and whether bypassed the closest hospital.

Subgroup	Survival rates (*n*)	Mean (sd) time intervals from the call to hospital admission (min)
High population density areas, bypassed the closest hospital	21.73% (1,615)	20.0 (6.5)
High population density areas, closest hospital	18.89% (2,017)	17.5 (5.8)
Low population density areas, bypassed the closest hospital	10.14% (759)	25.7 (9.9)
Low population density areas, closest hospital	10.50% (1,447)	22.0 (9.2)

Furthermore, areas with a low population density and prolonged interval from the call to hospital admission were associated with poor survival (10.14% in Table 3). Compared to cardiac arrests within the low population density areas (< 5,000 persons/km^2^), the likelihood of survival after OHCA was nearly twice as high (AOR = 1.89, 95% CI = [1.55–2.32]) within the high population density areas in the multivariate analysis. In addition, a one minute increase from the call to hospital admission was associated with a 2% reduction in survival (Table 2). Living in a low-density area was associated with an independent risk of delay in ambulance response and poor survival after OHCA in Sweden [17]. Accordingly, distribution of EMS units according to the population size may worsen survival inequality between the urban and rural areas. The interpolation map we created visualizing the estimated interval from the call to hospital admission (Fig 4) highlights the spatial inequality of EMS access in Kaohsiung. This map might aid future EMS allocation, like AED installation.

However, this observational study had several limitations. First, the major outcome of the study was survival to hospital admission. Other OHCA outcomes, like survival to hospital discharge or neurological outcomes were unavailable, thereby limiting the comparability of our results. Second, we failed to acquire data regarding patients’ health conditions before OHCA. Risk factors related to individual patients, like underlying comorbidities [38,39], might substantially influence survival. Third, the registry system only recorded the cardiac rhythm on each patient’s ED arrival and data regarding the initial cardiac rhythm or other Utstein elements was unavailable. Finally, we did not consider the impact of EMTs’ characteristics. Different EMTs’ capabilities and experiences might affect survival [40,41].

## Conclusions

On incorporating spatial and non-spatial factors in the study, we found that resuscitative efforts, such as AED or LMA use, and a short total EMS time interval improved OHCA outcomes in EDs. Female sex and events in public areas were associated with increased survival. The spatial heterogeneity of emergency medical resources between rural and urban areas might affect the survival rate and warrants in-depth exploration in future.
